# Chlorella Hot Water Extract Restores Collagen Production in Senescent Fibroblasts Through Reversal of miR-193a-5p-Mediated Translational Repression of COL1A1

**DOI:** 10.3390/nu18132163

**Published:** 2026-07-03

**Authors:** Sou Kageyama, Yusei Sato, Zenaida Aurea Krizza B. Escareal, Miharu Amano, Yuka Maejima, Yuji Kawabata, Takushi Namba

**Affiliations:** 1 Department of Marine Resource Science, Faculty of Agriculture and Marine Science, Kochi University, Oko-cho Kohashu Nankoku-shi, Kochi 783-8502, Japanb25m6h39@s.kochi-u.ac.jp (Y.S.); b25d6c03@s.kochi-u.ac.jp (Z.A.K.B.E.); b26m6h23@s.kochi-u.ac.jp (M.A.); m2620035@gunma-u.ac.jp (Y.M.); 2 Sunlife Company Limited, 101 Wakabadai, Meito-ku, Nagoya 465-0015, Japan; yk.001.0039@sunlife-rm.com; 3 Graduate School of Kuroshio Science, Kochi University, 2-5-1 Akebono-cho, Kochi 780-8520, Japan

**Keywords:** chlorella hot water extract, cellular senescence, type I collagen, miR-193a-5p, translational regulation, mitochondrial dysfunction, SOD2, dietary supplement, functional food

## Abstract

Background: Cellular senescence is accompanied by mitochondrial dysfunction and decline in type I collagen production, contributing to age-related tissue deterioration. However, the post-transcriptional mechanisms underlying senescence-associated collagen decline remain poorly understood. Methods: Here, we investigated the effects of chlorella hot water extract (CHWE) on mitochondrial function and collagen production in senescent human fibroblasts. Results: CHWE restored mitochondrial membrane potential, ATP production, and redox balance through upregulation of SOD2. Notably, CHWE increased collagen protein levels without altering COL1A1 mRNA, indicating post-transcriptional regulation. miRNA profiling across young, senescent, and CHWE-treated senescent fibroblasts revealed that miR-193a-5p was upregulated during senescence (1.73-fold) and normalized by CHWE treatment. Functional validation confirmed that miR-193a-5p mimic suppressed COL1A1 protein. These findings identify a senescence–miR-193a-5p–COL1A1 axis in which age-dependent miR-193a-5p accumulation represses collagen translation, and CHWE reverses this process. By simultaneously restoring mitochondrial bioenergetic capacity and relieving miRNA-mediated translational repression, CHWE promotes efficient collagen recovery in senescent cells through complementary mechanisms. Conclusions: This study reveals a translational regulatory mechanism of collagen decline during cellular aging and highlights CHWE as a functional food supplement and a potential multi-target agent for age-related tissue deterioration.

## 1. Introduction

Cellular senescence, characterized by irreversible growth arrest and impaired biosynthetic capacity, is a central driver of age-related tissue deterioration [1,2]. Senescent cells accumulate with age across diverse tissues, contributing to the functional decline of organs through disruption of extracellular matrix (ECM) homeostasis and chronic inflammation [3].

Mitochondrial dysfunction is both a cause and consequence of cellular senescence [4]. In senescent cells, mitochondrial membrane potential, ATP production, and antioxidant capacity decline, while mitochondrial reactive oxygen species (mtROS) accumulate [5,6]. The mitochondrial superoxide dismutase (SOD) 2 serves as the primary defense against mtROS, and its deficiency promotes senescence and aging phenotypes in vivo [7,8]. Critically, mitochondrial dysfunction impairs collagen biosynthesis in fibroblasts [9], linking mitochondrial integrity to ECM homeostasis [8].

Type I collagen, the most abundant structural protein in the body, declines substantially during cellular senescence [10]. Collagen expression is regulated at multiple levels, and post-transcriptional control plays a particularly important role: collagen mRNAs contain a conserved 5′ UTR stem-loop that governs translational efficiency via LARP6 binding, and 30–50% of collagen mRNAs are sequestered in translationally inactive pools [11]. Knockdown of the translational facilitator RNA helicase A reduces collagen protein without affecting mRNA levels [12,13], demonstrating that collagen output can be regulated independently of transcription.

MicroRNAs (miRNAs) are key mediators of such translational control. miRNAs suppress target gene expression by binding to the 3′ UTR of mRNAs, and their repression can reduce protein levels without altering mRNA abundance [14,15]. Several miRNAs directly target COL1A1: the miR-29 family exerts a greater impact on collagen protein than on mRNA [16,17], and miR-196a suppresses COL1A1 in keloid fibroblasts [18]. However, whether specific miRNAs are upregulated during senescence to suppress collagen translation, and whether such changes can be reversed by bioactive interventions, remain unexplored.

Chlorella hot water extract (CHWE), a polysaccharide-enriched extract from Chlorella microalgae containing nucleic acid-related substances, glycoproteins, peptides, and amino acids, has been consumed for decades as a nutrient-dense food and dietary supplement, and shown to promote cellular growth and protect against oxidative stress [19,20]. A recent study demonstrated that Spirulina polysaccharide restores mitochondrial function in aging fibroblasts via SOD2 upregulation [21], but the molecular mechanisms by which microalgae-derived extracts regulate collagen production—particularly through miRNA-mediated translational control—remain unknown. Here, we examined the effects of CHWE on mitochondrial function and collagen production using young and senescent human fibroblasts. We found that CHWE restored collagen protein without altering COL1A1 mRNA, and identified miR-193a-5p as a senescence-upregulated translational suppressor of COL1A1 whose expression is normalized by CHWE treatment.

## 2. Materials and Methods

### 2.1. Chlorella Hot Water Extract (CHWE)

Chlorella hot water extract (CHWE) was obtained as a spray-dried powder (CGF Powder W-10000; Sunlife Company Limited, Nagoya, Japan; manufactured by Vedan Biotechnology Corporation, Taichung, Taiwan). The extract was prepared by hot-water extraction from *Chlorella sorokiniana* biomass harvested during the logarithmic growth phase, cultivated under natural sunlight in Taiwan, followed by concentration and spray-drying. The product was standardized to an optical density (OD) value of ≥9500 at 260 nm. The proximate composition of the powder was determined by an independent accredited testing laboratory (Eurofins QKEN Co., Ltd., Munakata, Fukuoka, Japan; report No. 2025011504-001-2) using AOAC-compliant standard methods: protein content by the Kjeldahl titrimetric method; moisture, lipid and ash by gravimetric procedures; and total carbohydrate by calculation. The proximate composition is summarised in Table 1. CGF Powder W-10000 is a commercially available product marketed by Sunlife Company Limited for human consumption.

### 2.2. Cell Culture and Induction of Replicative Senescence

Human skin fibroblasts (NB1RGB; obtained from Riken BRC, Tokyo, Japan) were maintained in MEMα (Wako, Tokyo, Japan) containing 10% FBS, 100 U/mL penicillin, and 100 µg/mL streptomycin. Cultures were split at a 1:4 ratio every three days and kept at 37 °C under a 5% CO_2_ humidified atmosphere. Based on cumulative days in culture, cells were classified into two groups: young NB1RGB cells (8–20 days) and aging cells (60–70 days). The identical cell line previously employed by Machihara et al. was used here.

### 2.3. Cell Viability Assay

Cell viability was evaluated by the MTT (3-(4,5-dimethylthiazol-2-yl)-2,5-diphenyltetrazolium bromide) assay together with a cell count assay. Briefly, for the MTT assay, cells receiving the indicated treatments were incubated with MTT reagent (1 mg/mL) for 2 h. Subsequently, a mixture of isopropanol and HCl was added to yield final concentrations of 50% (*v*/*v*) and 20 mM, respectively, and the absorbance at 570 nm was recorded with a spectrophotometer (Infinite M200; TECAN, Tokyo, Japan).

### 2.4. Mitochondrial Membrane Potential (JC-1 Assay)

Mitochondrial membrane potential (ΔΨm) was assessed by JC-1 staining (Dojindo, Tokyo, Japan) in accordance with the manufacturer’s guidelines. JC-1 results are expressed as the ratio of fluorescence recorded at 535 nm/590 nm over that recorded at 485 nm/535 nm (aggregate-to-monomer fluorescence), acquired on a fluorescence microplate reader with the i-control 1.11 software (Infinite M200; TECAN, Tokyo, Japan). Fluorescence images were captured with a fluorescence microscope (BZ-X800; KEYENCE, Osaka, Japan) and processed with Adobe Photoshop elements 2024.

### 2.5. ATP Measurement

Intracellular ATP content was quantified with the CellTiter-Glo 2.0 assay kit (Cat. No. G9241; Promega, Madison, WI, USA) according to the manufacturer’s protocol. ATP output was normalized to total cellular protein.

### 2.6. Mitochondrial Superoxide Detection (mtSOX Staining)

Cells were first seeded into black- or clear-bottom culture plates. After the indicated treatments, cells were rinsed with phosphate-buffered saline (PBS) to remove residual medium and then stained for 10 min at 37 °C with 10 μM mtSOX Deep Red (Dojindo, Kumamoto, Japan) or MitoSOX Red (Invitrogen, Carlsbad, CA, USA). After an additional PBS wash, fluorescence was detected on a microplate reader using 535/670 nm or 535/590 nm filter pairs with the i-control 1.11 software (Infinite M200; TECAN, Tokyo, Japan). Corrected fluorescence intensity was calculated by subtracting the cellular autofluorescence from the signal of mtSOX Deep Red-treated cells. Representative fluorescence images were taken on a BZ-X800 fluorescence microscope (KEYENCE, Osaka, Japan).

### 2.7. Immunoblotting Analysis

Immunoblotting was carried out as described previously. The following primary antibodies were used: anti-SOD2 (Cell Signaling) and anti-β-actin (Sigma). All primary antibodies were applied at a 1:1000 dilution, with the exception of anti-β-actin, which was used at 1:10,000. HRP-conjugated secondary antibodies (anti-rabbit and anti-mouse) were obtained from Promega and used at a 1:5000 dilution.

### 2.8. Quantitative RT-PCR (qRT-PCR)

Total RNA was isolated and reverse-transcribed to cDNA using a commercial RT kit. The mRNA abundance of SOD2, COL1A1, and Hsp47 (SERPINH1) was determined by qRT-PCR and normalized to an internal reference gene with the 2^−ΔΔCt^ method. The primers used were (name: forward primer, reverse primer): *SOD2*: *5′-GGTGTGGCCGATGTGTCTAT-3′* and *5′-ACTTCCAGCGTTTCCTGTCT-3′*, *COL1A1*: *5′-CAAGAGGCATGTCTGGTTCG-3′* and *5′-TAGGTGATGTTCTGGGAGGC-3′*, *Hsp47*: *5′-GACCACCCCTTCATCTTCCT-3′* and *5′-ACTCGTCTCGCATCTTGTCA-3′*, *β-actin: 5′-GGACTTCGAGCAAGAGATGG-3′* and *5′-AGCACTGTGTTGGCGTACAG-3′.* For miRNA quantification, the Mir-X™ miRNA Quantification Kit (Takara Bio, Shiga, Japan) was used following the manufacturer’s protocol. qRT-PCR was carried out with miRNA-specific forward primers corresponding to the DNA equivalents of the mature miRNA sequences, paired with the kit-supplied mRQ 3′ universal reverse primer. Mature miRNA sequences were retrieved from miRBase (https://mirbase.org/) and converted to DNA: hsa-miR-98-5p, *5′-TGAGGTAGTAAGTTGTATTGTT-3′*; hsa-miR-129-5p, *5′-CTTTTTGCGGTCTGGGCTTGC-3′*; hsa-miR-143-5p, *5′-GGTGCAGTGCTGCATCTCTGGT-3′*; hsa-miR-193a-5p, *5′-TGGGTCTTTGCGGGCGAGATGA-3′*; hsa-miR-218-5p, *5′-TTGTGCTTGATCTAACCATGT-3′*; hsa-miR-338-3p, *5′-TCCAGCATCAGTGATTTTGTTG-3′*. miRNA expression was reported as absolute values.

### 2.9. Collagen Quantification

Collagen content was determined with a collagen quantitation kit (Cat. No. COL-001; Cosmo Bio, Tokyo, Japan) according to the manufacturer’s protocol. Collagen levels were normalized to total protein. Fluorescence was recorded on a fluorescence microplate reader (Infinite M200; TECAN, Tokyo, Japan) with a 360/485 nm filter pair.

### 2.10. miRNA Mimic Transfection

To assess the functional contribution of miR-193a-5p to COL1A1 regulation, fibroblasts were transfected with hsa-miR-193a-5p mimic (5′-UGGGUCUUUGCGGGCGAGAUGA-3′) or miRNA mimic Negative Control #1 using Lipofectamine RNAiMAX (Invitrogen, Carlsbad, CA, USA) following the manufacturer’s procedure. The miRNA mimics were sourced from the AccuTarget™ miRNA mimic library (BIONEER, Daejeon, Republic of Korea). Transfected cells were subsequently harvested for Western blot analysis of COL1A1 protein.

### 2.11. Bioinformatic Prediction of miRNA Target Sites

Candidate miRNA binding sites within the 3′ UTR of the COL1A1 transcript were predicted using bioinformatic tools. Based on these predictions, six miRNAs (miR-98-5p, miR-129-5p, miR-143-5p, miR-193a-5p, miR-218-5p, and miR-338-3p) were chosen as candidate COL1A1-targeting miRNAs for experimental verification.

### 2.12. Statistical Analysis

For comparisons between two groups, unpaired datasets were analyzed by either Student’s *t*-test or Welch’s *t*-test, depending on whether sample sizes and variances were comparable. When multiple compounds were tested within a single cell type, ANOVA was applied. For experiments in which two cell types were exposed to several compounds, two-way ANOVA was used and followed by Tukey’s HSD post hoc test. A *p* value below 0.05 was regarded as statistically significant. All analyses were carried out with Mac Statistical Analysis software Ver. 3.0 (Esumi, Tokyo, Japan).

## 3. Results

### 3.1. CHWE Is Non-Cytotoxic Across a Wide Concentration Range

**Figure 1:**
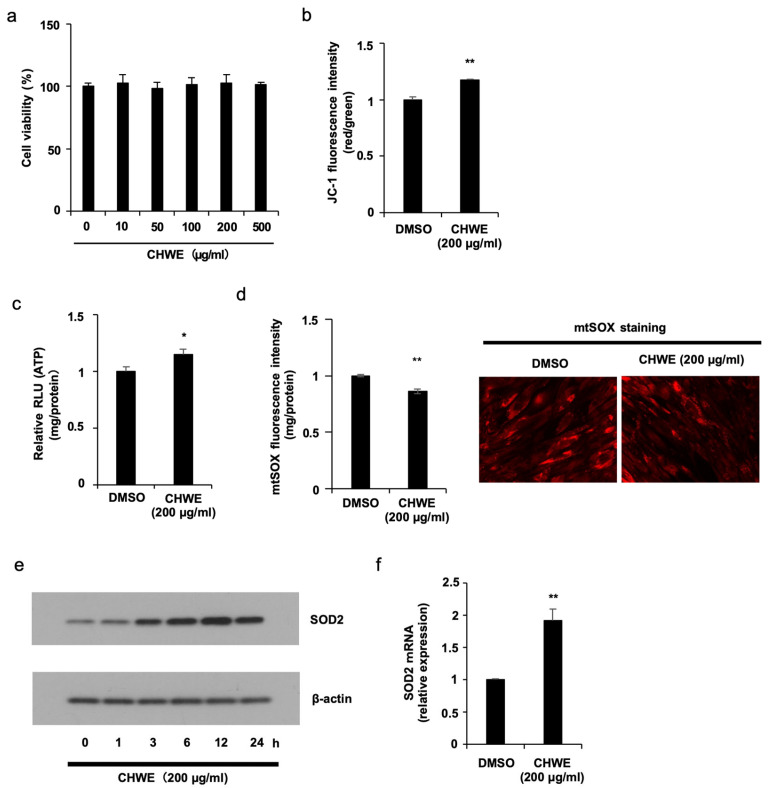
CHWE restores mitochondrial function in senescent fibroblasts. (**a**) Cell viability following CHWE treatment at indicated concentrations. (**b**) JC-1 red/green fluorescence ratio (mitochondrial membrane potential) in senescent fibroblasts treated with CHWE (200 µg/mL) vs. untreated senescent controls. (**c**) Relative ATP levels (RLU/mg protein). (**d**) mtSOX fluorescence intensity (mitochondrial superoxide) (left panel). Representative mtSOX fluorescence images (DMSO vs. CHWE) (right panel). (**e**) Time-course Western blot of SOD2 protein (0–24 h). (**f**) SOD2 mRNA by qRT-PCR. β-actin: loading control. *p* values were calculated using Student’s *t* test. * *p* < 0.05, ** *p* < 0.01 vs. untreated senescent control.

Prior to investigating the biological effects of Chlorella hot water extract (CHWE), we first assessed its potential cytotoxicity to establish a safe working concentration. Cell viability was measured following treatment with CHWE at 0, 10, 50, 100, 200, and 500 µg/mL. No significant change in viability was observed at any concentration tested (Figure 1a), confirming that CHWE is non-cytotoxic up to 500 µg/mL. Based on these results, 200 µg/mL was selected for all subsequent experiments.

### 3.2. CHWE Restores Mitochondrial Function in Senescent Fibroblasts

Mitochondrial dysfunction is a hallmark of cellular senescence and a known contributor to the decline of biosynthetic capacity in aging cells. We therefore investigated whether CHWE could restore mitochondrial function in senescent fibroblasts.

Mitochondrial membrane potential was assessed by JC-1 fluorescence. CHWE-treated senescent fibroblasts showed a significantly higher JC-1 red/green ratio than untreated senescent controls (Figure 1b), indicating restoration of the electrochemical gradient across the inner mitochondrial membrane. Consistent with this, cellular ATP production was also increased, suggesting recovery of oxidative phosphorylation capacity (Figure 1c).

To determine whether CHWE also reduces mitochondrial oxidative stress, we measured mitochondrial superoxide levels using mtSOX staining. CHWE treatment significantly decreased mtSOX fluorescence intensity (Figure 1d; left panel), and representative fluorescence images confirmed this reduction (Figure 1d; right panel). Together, these results demonstrate that CHWE restores key parameters of mitochondrial function—membrane potential, ATP output, and redox balance—in senescent fibroblasts.

### 3.3. CHWE Upregulates SOD2 Expression

Given the observed reduction in mitochondrial superoxide, we examined whether CHWE enhances the expression of SOD2, the primary mitochondrial antioxidant enzyme. Western blot analysis revealed a time-dependent induction of SOD2 protein over 24 h following CHWE treatment (Figure 1e). qRT-PCR confirmed that SOD2 mRNA was also significantly upregulated (Figure 1f). These data indicate that CHWE reduces mitochondrial superoxide at least in part through transcriptional upregulation of SOD2, although direct antioxidant activity of CHWE components cannot be excluded.

### 3.4. CHWE Restores Collagen Protein Levels in Senescent Fibroblasts

**Figure 2:**
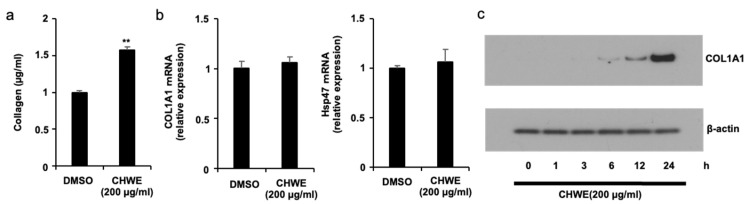
CHWE restores collagen production via a post-transcriptional mechanism. (**a**) Secreted collagen quantification (48 h) in senescent fibroblasts treated with CHWE vs. untreated senescent controls. (**b**) COL1A1 mRNA and Hsp47 by qRT-PCR (not significantly changed). (**c**) Time-course Western blot of COL1A1 protein (0–24 h). β-actin: loading control. COL1A1, β-actin, and SOD2 (Figure 1f) were detected from a single gel that was cut into separate sections and probed individually. Therefore, the β-actin loading control shown in Figure 1f and Figure 2c is identical (the same blot is presented in both panels). *p* values were calculated using Student’s *t* test. ** *p* < 0.01 vs. untreated senescent control.

Because senescent fibroblasts exhibit diminished collagen production, which contributes to age-related ECM deterioration, we next examined whether CHWE could reverse this decline. Quantification of secreted collagen after 48 h of treatment demonstrated that CHWE significantly increased collagen levels in senescent fibroblasts (Figure 2a).

### 3.5. Collagen Restoration Occurs Without Transcriptional Activation

To determine whether this increase was driven by transcriptional upregulation, we measured the mRNA levels of *COL1A1* and the collagen-specific chaperone Hsp47. Unexpectedly, CHWE did not significantly alter *COL1A1* mRNA and *Hsp47* mRNA expression level (Figure 2b), indicating that the collagen-restoring effect is not attributable to increased transcription.

In contrast, Western blot analysis revealed a time-dependent increase in COL1A1 protein of CHWE treatment (Figure 2c). This discrepancy—unchanged mRNA but elevated protein—suggests that CHWE promotes collagen production through a post-transcriptional mechanism, potentially at the level of translational regulation or protein stabilization. This finding prompted us to examine whether miRNAs, which can repress translation without affecting mRNA stability, mediate this effect.

### 3.6. miR-193a-5p Is Upregulated During Senescence and Reversed by CHWE

We hypothesized that senescence-associated changes in miRNA expression may suppress COL1A1 translation, and that CHWE may reverse these changes. Using bioinformatic target prediction, we identified six candidate miRNAs predicted to target the COL1A1 3′ UTR (miR-98-5p, miR-129-5p, miR-143-5p, miR-193a-5p, miR-218-5p, and miR-338-3p). Their expression was compared across three groups: young fibroblasts, senescent fibroblasts, and CHWE-treated senescent fibroblasts.

**Figure 3:**
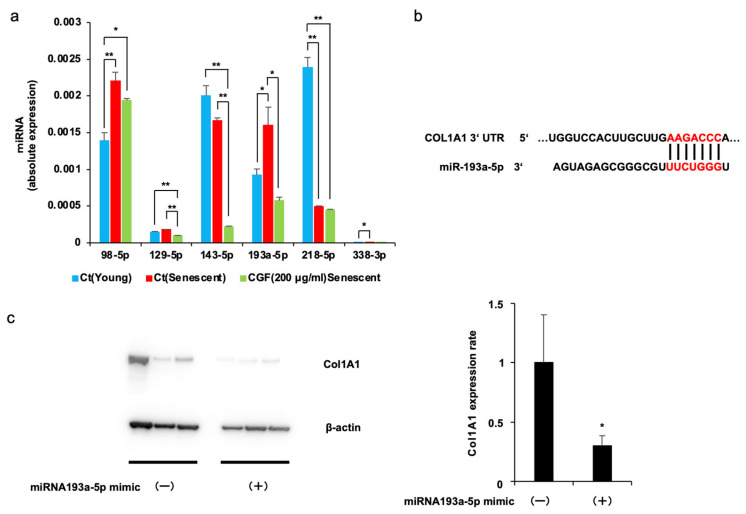
Senescence-dependent miR-193a-5p upregulation is reversed by CHWE, and miR-193a-5p suppresses COL1A1. (**a**) Expression of six candidate miRNAs across young, senescent, and CHWE-treated senescent fibroblasts. (**b**) Predicted miR-193a-5p binding site in the COL1A1 3′ UTR. (**c**) Western blot and quantification of COL1A1 protein following miR-193a-5p mimic (+) vs. negative control (-) transfection. *p* values were calculated via ANOVA following the Tukey-HSD test. * *p* < 0.05, ** *p* < 0.01.

qRT-PCR analysis revealed distinct expression patterns across the three groups (Figure 3a). miR-98-5p and miR-193a-5p showed the same overall response, being upregulated during senescence and reduced by CHWE, which identified them as the candidate miRNAs responsible for repressing COL1A1 translation in senescent fibroblasts. The magnitude of the CHWE-induced reduction, however, was substantially greater for miR-193a-5p than for miR-98-5p, so miR-193a-5p was predicted to be the main target through which CHWE relieves COL1A1 translational repression. Among the other candidates, miR-129-5p and miR-143-5p were unchanged during senescence but reduced by CHWE, miR-218-5p was decreased during senescence, and miR-338-3p, although upregulated in senescence and reduced by CHWE, was expressed at a very low absolute level. miR-193a-5p was therefore selected for functional validation.

### 3.7. miR-193a-5p Mimic Suppresses COL1A1 Protein Expression

Bioinformatic analysis identified a putative miR-193a-5p recognition sequence within the COL1A1 3′ UTR (Figure 3b). To functionally validate this predicted relationship, fibroblasts were transfected with a miR-193a-5p mimic and COL1A1 protein levels were assessed by Western blot. miR-193a-5p mimic markedly suppressed COL1A1 protein expression compared to the negative control mimic (Figure 3c). This result confirms that elevated miR-193a-5p is sufficient to suppress COL1A1 at the protein level.

## 4. Discussion

We showed that Chlorella hot water extract (CHWE) restores collagen production in senescent human fibroblasts through a post-transcriptional mechanism involving miR-193a-5p. CHWE increased COL1A1 protein without altering its mRNA, and we identified miR-193a-5p as a senescence-upregulated miRNA that suppresses COL1A1 translation and is normalized by CHWE treatment. In parallel, CHWE restored mitochondrial function through SOD2 upregulation.

### 4.1. Senescence-Associated Translational Repression of Collagen via miR-193a-5p

The central finding of this study is the identification of a senescence–miR-193a-5p–COL1A1 regulatory axis. The dissociation between unchanged COL1A1 mRNA and restored protein levels following CHWE treatment is consistent with the established paradigm that collagen expression is extensively regulated at the translational level [11,12]. Our three-group comparison revealed that miR-193a-5p is upregulated 1.73-fold during senescence and reversed by CHWE to levels below the young cell baseline, while functional validation confirmed that miR-193a-5p mimic suppresses COL1A1 protein to 0.34-fold. These data support a model in which age-dependent accumulation of miR-193a-5p contributes to collagen decline in senescent cells.

This finding adds a new dimension to the known repertoire of collagen-targeting miRNAs. Both the miR-29 family and miR-193a-5p repress COL1A1, but they become dysregulated in opposite directions in different pathological states [16,17]. In fibrosis, miR-29 is downregulated; the loss of this repression permits collagen overproduction. In senescence, by contrast, miR-193a-5p is upregulated; this gain of repression causes collagen decline. Thus, miRNA-mediated repression of collagen translation can be pathological either when it is lost (fibrosis) or when it is excessive (senescence), underscoring the context-dependent nature of collagen translational regulation and suggests that therapeutic strategies must account for the specific miRNA landscape of each pathological state.

### 4.2. CHWE as a Multi-Target Anti-Aging Agent

CHWE simultaneously restored mitochondrial function (membrane potential, ATP, mtROS) through SOD2 upregulation and reversed senescence-associated miR-193a-5p elevation to restore collagen production. Collagen biosynthesis is an energy-intensive process that requires functional mitochondria for the hydroxylation and folding of procollagen chains. By restoring mitochondrial bioenergetic capacity while concurrently relieving miR-193a-5p-mediated translational repression of COL1A1, CHWE may promote collagen production in senescent fibroblasts through two complementary mechanisms: providing the cellular energy required for efficient collagen biosynthesis and simultaneously de-repressing COL1A1 translation. This convergent action on both the energetic and regulatory prerequisites of collagen production may explain the robust restoration of collagen protein observed in this study. Whether these two pathways are mechanistically linked—for example, whether reduced oxidative stress secondarily normalizes the miRNA landscape—or operate independently remains to be determined. The convergence on SOD2 as a mitochondrial mediator parallels findings with Spirulina polysaccharide [21], suggesting that polysaccharide-enriched microalgal extracts may share a common mechanism of mitochondrial protection while exerting additional, extract-specific effects on post-transcriptional gene regulation.

### 4.3. Limitations and Future Applications

This study has several limitations. The direct binding of miR-193a-5p to the COL1A1 3′ UTR was not formally demonstrated and requires confirmation by a luciferase reporter assay, which is a priority for our ongoing work. Moreover, our functional validation relied on a gain-of-function (mimic) approach; a complementary loss-of-function experiment—inhibition of endogenous miR-193a-5p by antagomiR in senescent cells—would further establish causality and confirm that relief of miR-193a-5p repression underlies the collagen-restoring effect of CHWE.

In the present study, the biological effects of CHWE were examined using the water-soluble fraction obtained by hot-water extraction. CHWE was characterized at the level of proximate composition, but the specific chemical sub-fractions or active component(s) within this water-soluble fraction responsible for the observed effects were not isolated and remain to be identified through activity-guided fractionation. In addition, because CHWE is intended to be consumed orally as a dietary supplement, the in vitro concentration used in this study (200 µg/mL) cannot yet be directly equated with physiologically achievable levels in vivo. Although it is uncertain whether such a concentration is reached in the systemic circulation, comparable local concentrations may plausibly occur within the gastrointestinal tract following oral intake; the oral bioavailability and tissue distribution of the active constituents will require evaluation in future nutritional and pharmacokinetic studies.

Finally, the present findings were obtained in a replicatively senescent NB1RGB fibroblast model. Their generalizability should be confirmed in primary fibroblasts from aged human donors and, ultimately, in in vivo models, in which the senescence–miR-193a-5p–COL1A1 axis and the effect of orally administered CHWE on dermal collagen could be assessed directly.

From a translational perspective, the identification of miR-193a-5p as a senescence-associated suppressor of collagen translation opens therapeutic possibilities beyond conventional transcription-based approaches. Because CHWE is non-cytotoxic, derived from a food-grade source, and targets both mitochondrial dysfunction and miRNA-mediated collagen decline, it represents a promising candidate for nutraceutical or cosmeceutical applications aimed at counteracting age-related tissue deterioration. Furthermore, miR-193a-5p itself may serve as a biomarker of collagen-producing capacity in aging tissues and a potential target for antagomiR-based interventions in age-related fibroblast dysfunction.

## 5. Conclusions

Chlorella hot water extract (CHWE) restored collagen production in senescent human fibroblasts through a post-transcriptional mechanism involving SOD2-mediated mitochondrial recovery and reversal of miR-193a-5p-dependent translational repression of COL1A1. By identifying miR-193a-5p as a senescence-associated suppressor of collagen translation and demonstrating that CHWE can reverse this regulation, the present study highlights a novel therapeutic axis for combating age-related extracellular matrix decline and supports the development of microalgal extracts as multi-target anti-aging agents.

## Figures and Tables

**Table 1:** Proximate composition of CHWE.

Component	Amount (g/100 g)	Analytical Method
**Moisture**	1.3	Gravimetric
**Protein**	68.7	Kjeldahl (titrimetric)
**Lipid (crude fat)**	0.4	Gravimetric
**Carbohydrate**	8.8	Calculated
**Ash**	20.8	Gravimetric

## Data Availability

All data needed to evaluate the conclusions in this paper are presented in the paper. Additional data related to this paper may be requested from the author.
